# An Aggressive Angiomyxoma Presenting as a Vaginal Wall Carbuncle in a 31-Year-Old Female: A Case Report

**DOI:** 10.7759/cureus.75512

**Published:** 2024-12-10

**Authors:** Eman M Keshk, Saud O Alafghani, Muhammad Usman Tariq, Ammara Kashif, Abdullah Alsulaiman

**Affiliations:** 1 Histopathology Department, Ahmed Maher Teaching Hospital, Cairo, EGY; 2 Histopathology Unit, Laboratory Department, Al Hada Armed Forces Hospital, Taif, SAU; 3 Genetics Unit, Laboratory Department, Al Hada Armed Forces Hospital, Taif, SAU

**Keywords:** aggressive angiomyxoma, carbuncle, desmin, estrogen receptors, infiltrative, vagina

## Abstract

An aggressive angiomyxoma (AA) is a rare soft tissue neoplasm of the lower female genital tract. The incidence of vaginal involvement is low, so it is commonly misdiagnosed as a Bartholin cyst, lipoma, and abscess, among others. This is a case of a 31-year-old female patient presenting with anterior vaginal wall swelling measuring 1 x 1 cm, clinically diagnosed as vaginal carbuncle. Histological and immunohistochemical examination of the lesion showed an AA. Some AAs recur locally, while distant metastasis is rare. The tumor is infiltrative with a characteristic histologic picture. The tumor is positive for high mobility group A2 (HMGA2), estrogen receptors (ERs), a cluster of differentiation 34 (CD34), and desmin immunomarkers. Due to the infiltrative nature of the tumor, surgical margins are commonly positive.

## Introduction

A deep angiomyxoma is rare, a locally infiltrative tumor, which occurs in the pelvis and perineum of women, in their third to fourth decades [1]. This tumor was first described by Steeper and Rosai as a slowly growing, low-grade neoplasm of the pelvis and vulvoperineal region [2]. Due to its anatomical distribution, it might be mistaken for labial and Bartholin cysts, abscesses, and other soft tissue lesions [1]. Clinically, tumors are variable-sized and might reach more than 10 cm in diameter, causing cosmetic disfigurement [3].

Aggressive angiomyxomas (AAs) have a high incidence of local recurrence (30%) that sometimes occurs many years after the initial diagnosis, with the not-unusual occurrence of multiple recurrences, which may be destructive [3]. Metastatic disease from the tumor is very unusual [4].

Grossly, tumors are soft and gelatinous with ill-defined margins [5]. Histologically, it is a hypocellular infiltrative neoplasm formed of bland spindle cells with bipolar cytoplasmic processes and no remarkable cytologic atypia, set in an abundant myxoid stroma. Numerous variable-sized blood vessels that are usually thick-walled and hyalinized are seen, with a surrounding collection of smooth muscle fibers (myoid bundles) and collagen bundles [6]. Tumor cells are immunoreactive for desmin and actin, especially the myoid bundles. Stromal spindle cells are positive for HMGA2, ER, PR, and CD34 [6].

Tumors most commonly show abnormalities of 12q15. Some cases reveal rearrangement of HMGA2, a deoxyribonucleic acid (DNA) architectural factor important for transcriptional regulation, which is also aberrantly expressed in other tumors (e.g., uterine leio­myoma, lipoma) with similar translocations [7].

Differential diagnose includes plexiform neurofibroma, angiomyofibroblastoma, myxoid smooth muscle tumors, superficial angiomyxoma, vulvar hypertrophy, myxoma, and myxoid liposarcoma [4]. Treatment is wide local excision, and a 1 cm negative margin is considered optimal [6].

## Case presentation

A 31-year-old female patient presented to the Gynecology Department at Al Hada Military Hospital, Saudi Arabia, with vaginal swelling and discharge, clinically diagnosed as vaginal wall carbuncle. Her past medical history was unremarkable.

**Figure 1:**
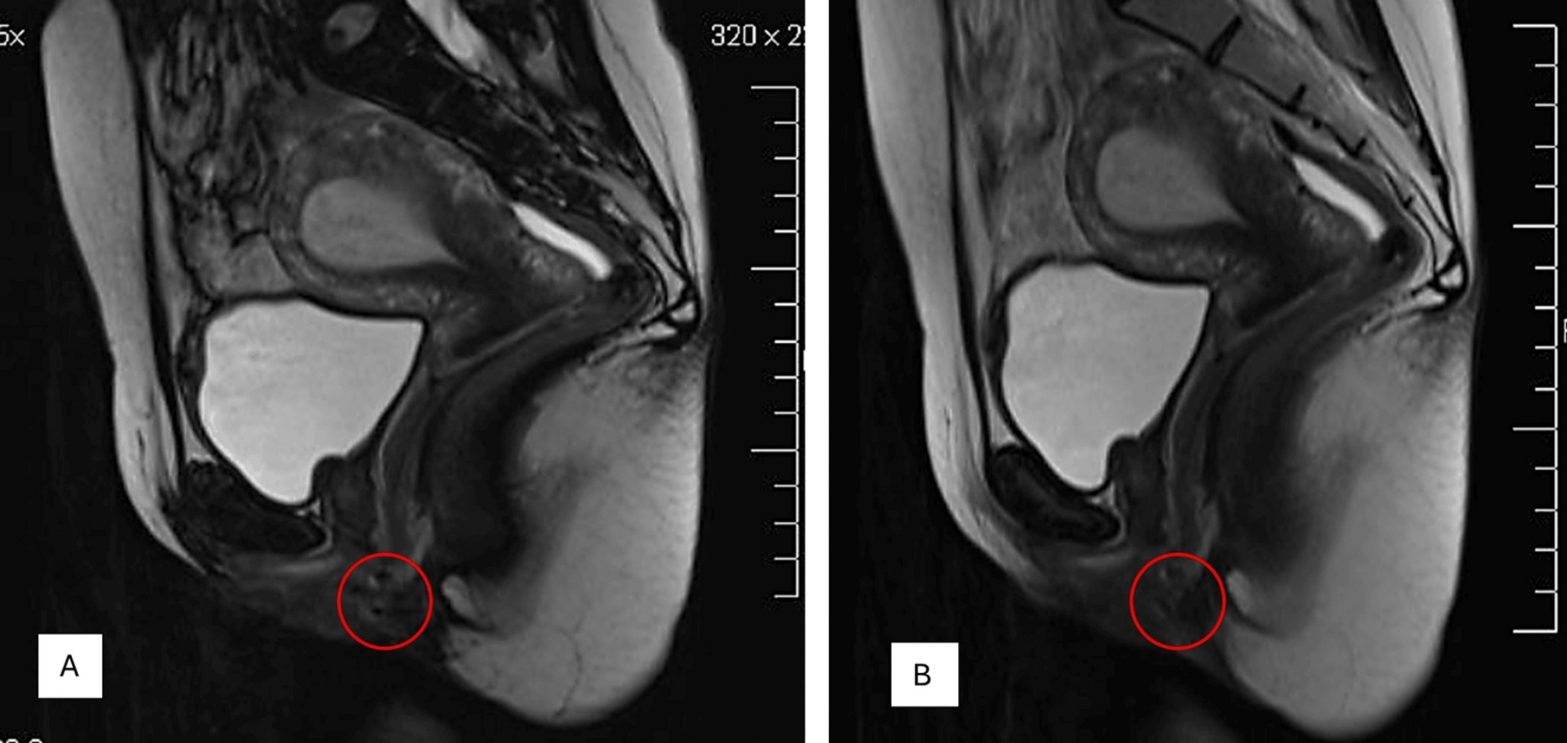
(A and B) Magnetic resonance image (MRI) showing a small lower vaginal and para-urethral hypodense lesion. Red circles highlight the lesion.

Magnetic resonance imaging (MRI) showed a lower and anterior vaginal lesion with high T2 signaling (Figure 1).

Afterward, surgical excision was done. The lesion was received at the hospital's Histopathology Laboratory. Grossly, the lesion was soft greyish, mucoid, and translucent, focally covered by vaginal mucosa, measuring 1.5 x 1.5cm.

**Figure 2:**
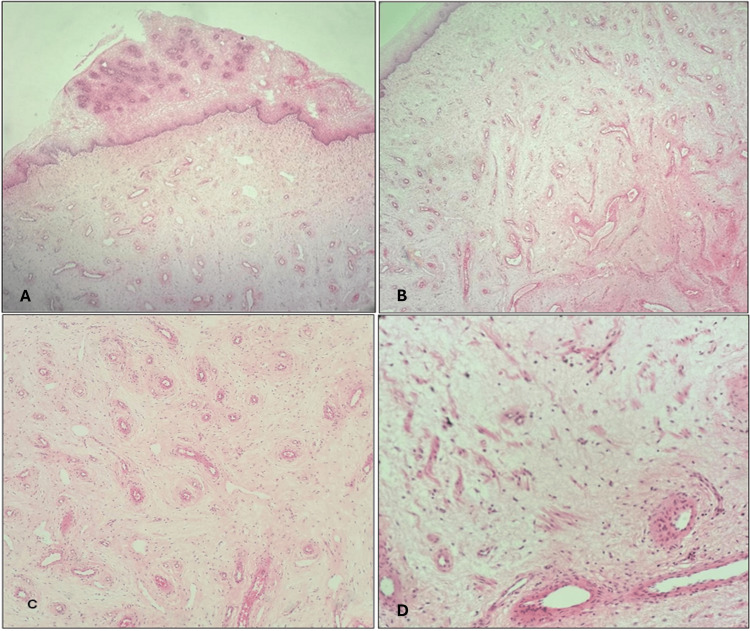
(A&B) Vaginal wall with submucosal aggressive angiomyxoma, showing proliferating blood vessels and hypocellular myxoid spindle cell stroma. H&E, X40 and X100, respectively. (C) Tumor with small to medium-sized hyalinized blood vessels, bland stromal spindle cells and mucin. H&E, X100. (D) Tumor stromal mucin with scattered myoid bundles. H&E, X200.

**Figure 3:**
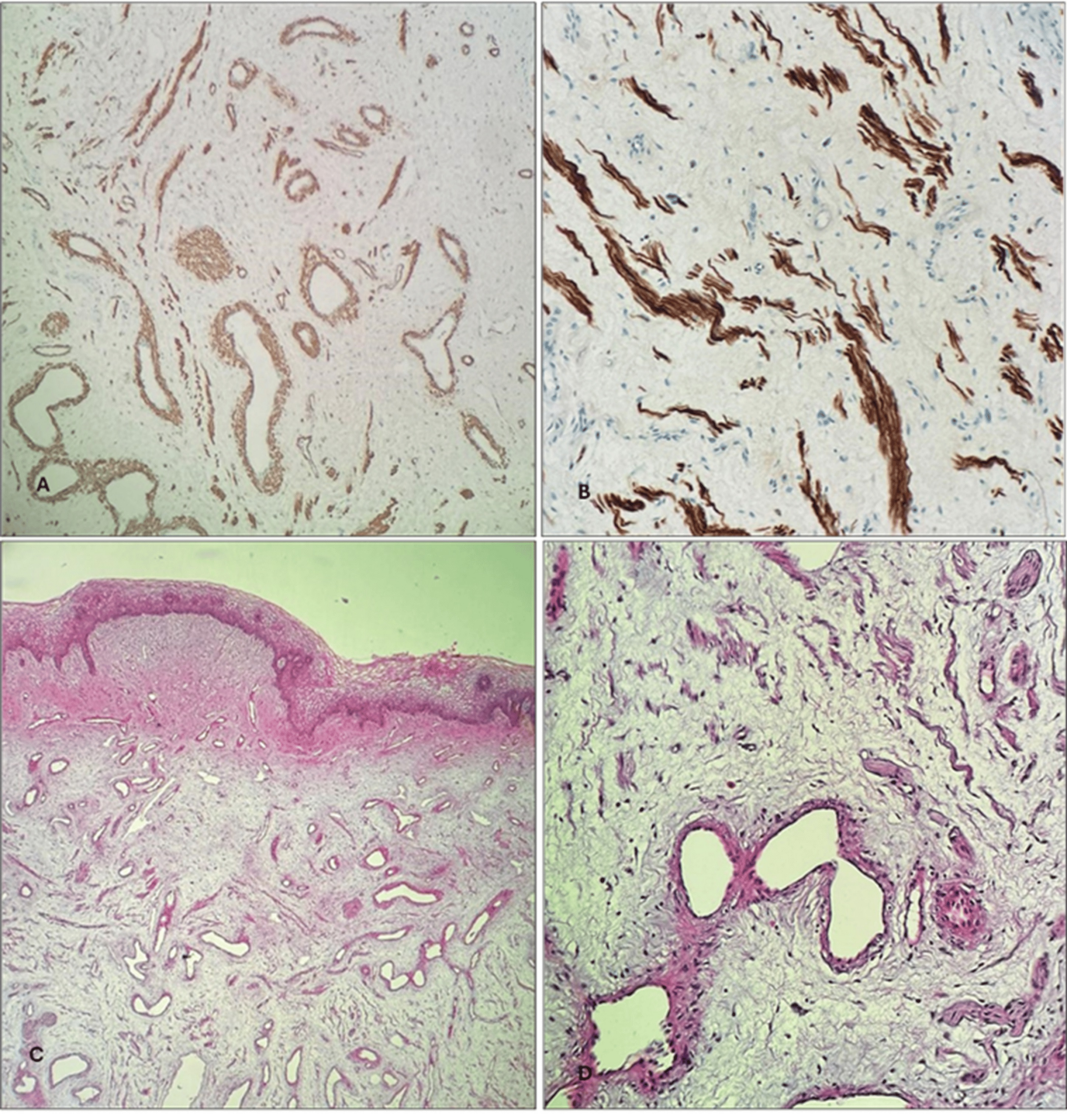
(A) Desmin stain showing positive staining in blood vessels and stromal myoid bundle. Desmin, X100. (B) SMA is showing positive staining in stromal myoid bundles. SMA, X200. (C&D) Alcian Blue stain highlighting stromal mucin. Alcian Blue, X100 and X200, respectively.

Histologically, a neoplastic lesion was detected, which was poorly circumscribed hypocellular, formed of bland spindle cells with bipolar cytoplasmic processes set in abundant myxoid stroma (Figure 2); the latter was positive for Alcian blue special stain (Figure 3). Variable-sized blood vessels with focal mural hyalinization were detected, as well as proliferating stromal myoid bundles (Figure 2). The tumor reached surgical margins.

Desmin and smooth muscle actin (SMA) were positive as well, especially in stromal myoid bundles (Figure 3).

**Figure 4:**
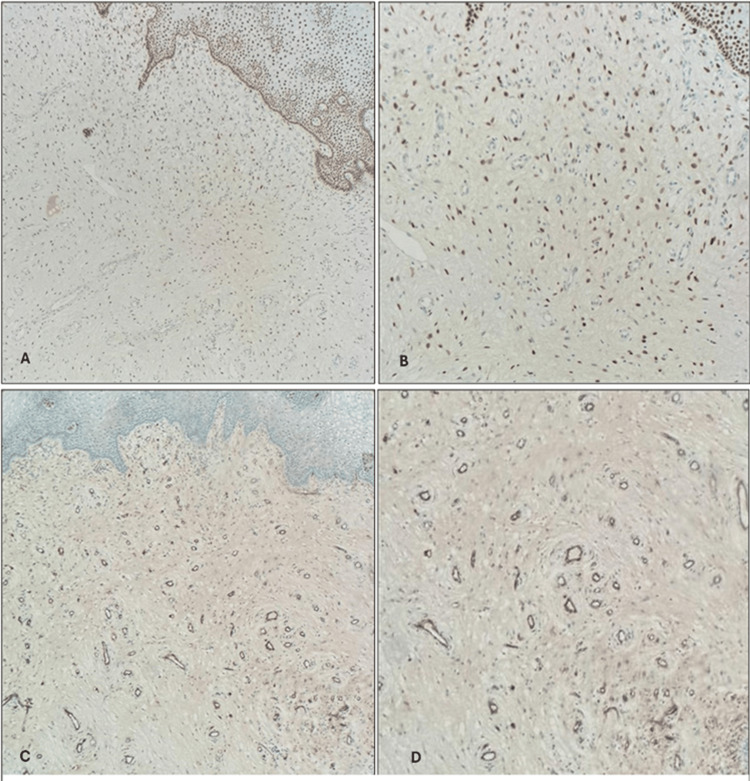
(A and B) ER immunostains showing nuclear staining of the tumor cells. ER, X100 and X200, respectively. (C and D) CD34 immunostain showing focal positivity of the tumor cells. CD34, X100.

Positivity for estrogen receptors (ER) and focal positivity for CD34, with positive internal control of blood vessels, can be also detected (Figure 4). Based on the histopathology and immunohistochemistry, a diagnosis of vaginal wall AA was made.

**Figure 5:**
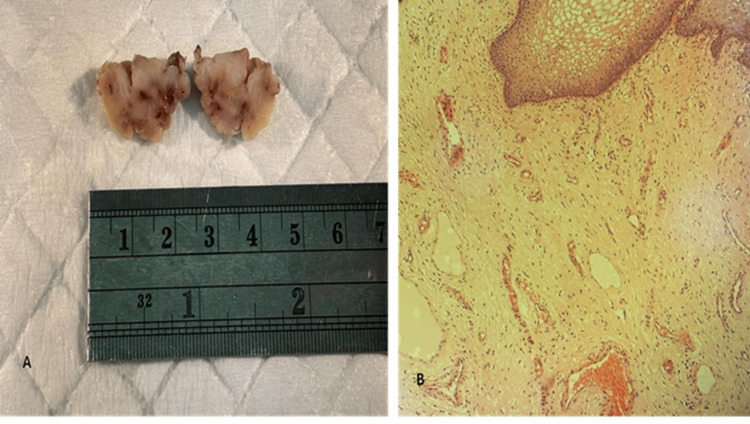
(A) Widening of the surgical margin gross picture. (B) Microscopic examination of margin widening shows involvement by neoplastic tissue. H&E stain, X100

In view of the histological diagnosis of an AA, the surgical margin was revised, However, the revised margin was also involved by the tumor (Figure 5).

## Discussion

We diagnosed a case of vaginal AA in a 31-year-old female patient, a rare tumor with common clinical misdiagnosis, especially on this site. Most cases occur in perimenopausal females [8], which usually present as accidentally discovered, slow-growing vulvar or perineal masses that may go unnoticed for years before being correctly diagnosed [9]. The tumor shows positive staining for ER and progesterone receptor (PR), which play a role in tumor evolution, especially in childbearing age [8]. Our case expressed positive nuclear staining for ER, in stromal spindle cells.

Grossly, AA is used to have smooth borders and infiltrative margins into the host’s tissues [10], while histologically, the tumor consists of a mixed population of hypocellular spindle cells, spread in a loose myxoid matrix with interspersed collagen bundles, as well as variable-sized blood vessels, which may be hyalinized [8]. 

AA has a recurrence rate of about 30% and usually has the same histological picture when recurring [11]. Tumor differential diagnosis includes angiomyofibroblastoma, cellular angiofibroma, myxoma, and myxoid liposarcoma, all of which can be differentiated on radiological and histological grounds [12,13]. Treatment is mainly surgical excision, with a 1 cm negative margin, which is considered optimal. However, it might represent a surgical challenge [14]. The extent of surgical excision must be weighed against the risks, especially in patients of childbearing age [8], so some authors suggested treatment with gonadotropin-releasing hormone agonists as a neoadjuvant treatment to decrease the tumor size before surgery or as an adjuvant treatment to prevent recurrence in tumors, which are ER- and PR-positive [11].

Radiation therapy has been described as an alternative to resection in advanced disease or as an adjuvant treatment for recurrent tumors [11]. Watchful waiting has been suggested as a treatment option in some cases, where surgery carries more risks than benefits [15]. Clinical and radiological examinations are usually required after surgery for a follow-up due to the high incidence of tumor recurrence [10].

## Conclusions

This case report represents an AA presenting as a vaginal wall carbuncle. This is a rare tumor and must be considered in the differential diagnosis of any lower female genital tract masses. Patients should be informed about the difficulty of having clear margins and the possibility of tumor recurrence.
